# Reviewing Ligand-Based Rational Drug Design: The Search for an ATP Synthase Inhibitor

**DOI:** 10.3390/ijms12085304

**Published:** 2011-08-17

**Authors:** Chia-Hsien Lee, Hsuan-Cheng Huang, Hsueh-Fen Juan

**Affiliations:** 1 Graduate Institute of Biomedical Electronics and Bioinformatics, National Taiwan University, Taipei 106, Taiwan; E-Mail: r99945042@ntu.edu.tw; 2 Institute of Biomedical Informatics and Center for Systems and Synthetic Biology, National Yang-Ming University, Taipei 112, Taiwan; 3 Institute of Molecular and Cellular Biology and Department of Life Science, National Taiwan University, Taipei 106, Taiwan

**Keywords:** pharmacophore, quantitative structure-activity relationships, ATP synthase ligands

## Abstract

Following major advances in the field of medicinal chemistry, novel drugs can now be designed systematically, instead of relying on old trial and error approaches. Current drug design strategies can be classified as being either ligand- or structure-based depending on the design process. In this paper, by describing the search for an ATP synthase inhibitor, we review two frequently used approaches in ligand-based drug design: The pharmacophore model and the quantitative structure-activity relationship (QSAR) method. Moreover, since ATP synthase ligands are potentially useful drugs in cancer therapy, pharmacophore models were constructed to pave the way for novel inhibitor designs.

## 1. Introduction

Previously, new drugs were found only by a trial and error approach. Potentially, anything deemed to be of medicinal value in a specific disease could be tested on patients to determine its efficacy [1]. As the science of chemistry advanced, researchers started to purify the active compounds in herbal preparations known to have medicinal properties, and deduce the structures of these active compounds [1]. The science of drug design progressed further with advances in molecular biology and biochemistry, which elaborated the concepts of genes and ligand-receptor relationships [2]. The most important breakthrough came with the emergence of genomics, proteomics and the development of bioinformatics and chemoinformatics. The concept and techniques of large-scale screening, which can be applied to identify disease-related genes and proteins, were first introduced in genomics and proteomics [3], as bioinformatics [4] and chemoinformatics [5] further sought to uncover methods for large-scale data processing and *in silico* experimentation.

The goal of drug design is to select a target in the etiology of a disease and find one or more compounds which interacts with that target [6], and in doing so activates or blocks the given target [2]. Ideally, the resultant changes in target protein activity will go on to influence a series of reactions and lead to an improvement in the clinical outcome [7].

After a suitable target has been identified, the drug design process can begin. If reliable information on the 3-D structure and active sites of the target protein can be obtained from X-ray crystallography, nuclear magnetic resonance, or 3-D structure databases, and incorporated into a computer model, compounds binding to the target can be designed [8]. This approach is known as “structure-based drug design”. Frequently used techniques in this approach are docking and molecular dynamics simulation [9]. Potent ligands can be found by screening a molecule database with docking software [10]. Molecular dynamics simulation can be useful to ascertain not only how a molecule interacts with the target protein, but also to determine some other properties of the molecule itself, such as membrane permeability [11,12].

In some cases, usually in which data pertaining to the 3-D structure of a target protein are not available, drug design can instead be based on processes using the known ligands of a target protein as the starting point. This approach is known as “ligand-based drug design”. Molecular similarity approaches, quantitative structure-activity relationships (QSAR) and pharmacophore models are frequently used methods in the ligand-based drug design process [13]. By using the molecular fingerprints of known ligands, databases can be screened to find molecules with similar fingerprints [14]. Common structural features of ligands can be found using pharmacophore modeling, which can then be used to screen for molecules with these features [15]. To predict the activity of a novel molecule, models can be built with QSAR [16]. While a pharmacophore model may only indicate the activity-conferring features of an active ligand, the relationship between chemical or physical properties of ligand and biological activity can be more fully explored using the QSAR model.

This review will focus on QSAR and pharmacophore modeling and elaborate on their fundamental concepts, workflows for building models, and their applications. Pharmacophore models of the ATP synthase beta subunit-binding ligands selected from existing literature are also discussed here as an illustration.

## 2. Pharmacophore

The term “pharmacophore” was first defined by Ehrlich as: “a molecular framework that carries the essential features responsible for a drug’s biological activity” [17]. It follows from this definition that a pharmacophore defines the necessary features that an active ligand should possess. Generally, feature type, position, and direction of an active ligand would be encoded into a pharmacophore model, along with possible steric constraints of the active compound [18].

A 3-D pharmacophore would reflect how key amino acids are positioned in the active site of a target protein [19]. For example, a key amino acid residue which acts as a hydrogen-bond acceptor should be in the vicinity of a hydrogen-bond donor feature in the pharmacophore model, accounting, in part, for the protein-ligand interaction. Once a ligand binds to the target protein in the correct conformation and interacts with key amino acid residues, the conformation of the protein may be changed or become locked, depending on the mechanism of ligand-protein interaction [2].

A pharmacophore model can be generated from a set of known ligands. However, data pertaining to 3-D protein structure or protein-ligand complexes combined with information on active sites can also be used to model a pharmacophore [20]. By studying the binding site, possible interactions between the active compound and the protein can be inferred, and pharmacophore models can be built from data on target protein structure.

Pharmacophore models are widely used to elicit specific inhibitors of disease-related proteins, including G-protein coupled receptors, enzymes, and ion channels [21]. It is also used with other drug discovery methods, as has been described in the “Applications” subsection.

### 2.1. Construction of a Pharmacophore Model

The detailed workflow of pharmacophore model construction will depend on the software package one uses. However, the general procedure can be summarized as follows [18]: Ligands are first selected and their usability confirmed. If a 3-D pharmacophore model is being constructed, sets of conformers, or conformations of a ligand, are generated. According to IUPAC, conformations are defined as the spatial arrangement of the atoms affording distinction between stereoisomers [22]. The conformers, or the 2-D structures if a 2-D pharmacophore model, which define only the required atom types and their connectivity [23] being built, are then used to determine features common to the selected ligands required to build the pharmacophore model. In general, more than one pharmacophore model is generated from the selected ligand set. Ranking and validating the model is the final step in pharmacophore model construction. Most pharmacophore modelers conduct the whole workflow with the help of a chemoinformatics software package, since all the required tools are found in this type of package.

The first step in pharmacophore modeling is to assemble an active compound set, usually from literature searches and molecular database querying. The researchers can use all databases available, including commercial, in-house, or public databases. A consistent threshold to define a compound active should be applied while searching active compounds from multiple sources. Activity levels of the compounds are not necessary if it is not necessary to build a pharmacophore-based QSAR model subsequently. After a set of molecules is selected, ensuring that all selected molecules exert their biological effects via the same mechanism is another important issue which should be considered before continuing pharmacophore model construction [18]. The structures of the molecules should be confirmed after checking their biological mechanism. In some cases, the chemical structures are manually sketched in a software package, which is an error-prone process. Even if the chemical information comes from a well-known database, a modeler should be cautious since such data is not always accurate. The pharmacophore model describes the interaction between the active site of a target protein and a ligand [19]; therefore the use of data pertaining to different active sites on the target would produce an entirely spurious pharmacophore model. Even if the pharmacophore elucidation software could generate results from such input, the resulting model would be meaningless.

If a representative 3-D pharmacophore model is to be built, a reasonable conformation set should be generated prior to alignment. The quality of the output set of the conformation generator, including such considerations as coverage and energy of the conformers, impacts on the quality of generated model [24].

The next step is to build the pharmacophore model from the common features found among the ligands. To find common features, the ligands are aligned, or superimposed with the assistance either of a conformer database of the relevant ligands or of an on-the-fly conformation generator [20]. After the alignment step is completed, the pharmacophore models are generated by a pharmacophore elucidation algorithm. More than one output model can often be obtained from this procedure, and selecting the best ones from the results is an important task [25]. This is usually assisted by the scoring function contained within the pharmacophore-building software. Generally, the model with the highest score is the most representative member of the output model set.

The best models are subjected to the validation process. If the receptor-ligand binding mechanism is clearly understood, this information can be utilized to validate the pharmacophore model, since the pharmacophore model can sometimes reflect the 3-D structure of the binding site, as previously stated [19]. If a pharmacophore model conflicts with previously established binding mechanisms, the modeler should consider rejecting it, or try to account for the discrepancies between documented binding data and the newly built pharmacophore model. A pharmacophore model should always be validated by ligands that are not included in the training set despite whether previously known binding mechanisms exist or not. If a pharmacophore model cannot pass this validation, it should be rejected and the modeling process reviewed.

### 2.2. Applications of Pharmacophore Models

Pharmacophore models are often employed in virtual screening processes. For example, Mustata *et al*. discovered novel Myc-Max heterodimer disruptors [26] by using a pharmacophore model generated using known disruptors. In this study, the authors performed the virtual screening with the pharmacophore model after it was validated by using a non-training molecule set. Bioassays were then conducted to check the potency of the screened molecules.

Another example of the use of pharmacophore models in drug discovery is the identification of novel PPARγ partial agonists by Petersen *et al.* [27]. The authors found that full agonists at the PPARγ had unwanted side effects, as previous research had indicated [28], so it was a priority to find new compounds which could act as partial agonists at PPARγ. A pharmacophore model was built from a collection of known partial agonists, and it was validated with a newly discovered partial agonist. After inspecting the hit list generated by virtual screening with the pharmacophore model, methyl oleanonate, a derivative of oleanonic acid, was selected because oleanonic acid can be easily obtained. Purification and bioassay were conducted after its potential to act as a partial agonist was confirmed by docking simulation.

In addition to the uses described above, pharmacophore models can be used as filters to reduce false positives, either during preprocessing of a molecular database before submission to another virtual screening procedure [29], or to filter the results of other virtual screening processes [30].

An example of the use of a pharmacophore model as a preprocessor can be found in the Chk-1 kinase study by Lyne *et al.* [29]. In this research, the authors preprocessed the molecule database using molecular weight and rotational bond limits and a user-defined 3-D pharmacophore model. The remaining ligands were then docked into the binding site of the kinase and scored by a scheme defined by the authors. The potencies of the resulting molecules were tested in a Chk-1 kinase assay, an *in vitro* bioassay protocol.

Pharmacophore models can also be used to process the output of virtual screening protocols, as shown in the research of Peach *et al.* [30]. Although the pharmacophore model in this study was generated manually from receptor binding site data obtained from literature and 3-D structure database searches, it aptly demonstrated the way in which a pharmacophore model can be used as a filter. In this research, the authors supported the validity of their method with three examples. First, the pharmacophore model was constructed from 3-D receptor-ligand complexes and from literature data. A docking simulation was performed, with all output tested by the pharmacophore models. During this process, a molecule is only recognized as a hit if it matches with all generated pharmacophore models. This approach performed better than scoring functions provided by the docking algorithms used here. The value of this approach, in contrast to the pharmacophore preprocessor method, is that the time-consuming docking simulation does not need to be repeated if the pharmacophore model is subsequently changed [30].

## 3. QSAR

QSAR, which stands for “quantitative structure-activity relationships”, is a method that relates chemical structure to biological or chemical activity using mathematical models [31]. If the activity of a set of ligands can be determined, a model can be constructed to describe this relationship. Unlike a pharmacophore model, which encodes only the essential features of an active ligand, the QSAR model allows one to determine the effect of a certain property on the activity of a molecule. For example, the QSAR model may reveal a property to have a highly negative, or alternatively a weak positive effect on ligand activity. Such information is not available using a pharmacophore model.

Quantifying the structure and activity of a ligand is important in the modeling process. Structure quantification is not a trivial problem, since a structure cannot be represented by a mere value. Instead, a set of properties, usually known as the “descriptors”, is computed from the structure and used to quantify it. By using structural descriptors as independent variables and activity as a dependent variable, a model can be built to describe the relationship between the two [32].

After a QSAR model is built and validated, it can predict the biological activity of novel molecules from their structural properties. A QSAR model can also screen potentially active molecules from a database, as described in the section on applications of the technique. Because the QSAR model can incorporate a wide range of different variables, be it physical, chemical or biological, it can also be utilized in industries apart from drug design [33], such as toxicology [34], food chemistry [35], and other fields.

### 3.1. Building a QSAR Model

The process of constructing a QSAR model can be summarized as follows [36]: First, ligands and their activities are collected. Descriptors are calculated and selected before a mathematical modeling method is chosen and the ligand data are then used to construct the QSAR models. After the models are completed, they are tested by internal and external validation procedures. Only then can a QSAR model be used in any practical applications, such as predicting the activity of a novel compound.

As is the case when building a pharmacophore model, the active ligand set must be gathered from molecular databases or from literature searches before QSAR modeling begins. The process requires not only the collection of ligand structures but also of their activities. Generally, IC_50_s (half maximal inhibitory concentration) [37], EC_50_s (half maximal effective concentration) [38], and *K*_i_ values (inhibition constant) [39] are commonly used to quantify drug activity. However, the quantification of ligand activity as used in QSAR is not limited to pharmacokinetic parameters. Other activity indexes can also be incorporated into model depending on the phenomena one wishes to predict. For example, Chen *et al.* used corneal permeability coefficient as the activity index to construct a model that predicted the efficiency of drug delivery in various eye drop medications [40]. In addition to structure verification as described in the section on pharmacophore model construction, ligand activity data should also be checked. All activity data should come from the same experimental procedure or assay, and it is preferable if the data comes from the same laboratory, and even the same researcher [32].

Before a QSAR model can be built, ligand structure descriptors should be ascertained or calculated. Some descriptors obtained directly from data sources or calculated using simple arithmetic operations take into account the specific number of atoms, molecular chain length, molecular mass, .*etc*. However, other descriptors may require complex computation, for example pharmacophore-based descriptors [41], molecular field descriptors, which are derived from the interaction of probes and molecules and used in CoMFA and CoMSIA [42,43], and spectral descriptors derived from the IR spectrum of the ligand [44]. It is important that the descriptors are related to the biological or chemical activity which the model will be used to predict [45]. In other words, if a descriptor is not related to activity, one should avoid incorporating the descriptor into the modeling process.

After the activity index (the dependent variable) and descriptors (the independent variables) are prepared for each ligand, a variable selection method and a modeling method can be selected, and a model is built. The selection process should avoid redundant descriptors, and then a model is built using the selected descriptors [46]. If two descriptors represent a similar biological or chemical parameter, one of them should be disregarded. In order to select descriptors, genetic algorithms [47,48], principle component analysis [49], artificial neural networks [50] and *k*-nearest neighbor [51] approaches can all be used. If a linear model is assumed, some conventional statistical methods, such as the partial least squares method and multiple linear regression [16] can be used. If a nonlinear model is preferred on the other hand, machine learning methods like artificial neural networks [52] or support vector machines [53] can be applied.

The main differences among the frequently used QSAR algorithms reside in their means of descriptor generation. For example, most QSAR algorithms, like CoMFA [42], CoMSIA [43], CoMMA [54], and HypoGen [55], use similar linear statistical models to explore the relationship between activity and descriptors, which are calculated by different processes. In CoMFA and CoMSIA, pre-aligned molecules are put onto a grid, or lattice. The descriptors are calculated by the interaction of the molecule and a probe is placed at each intersection of the lattice. The differences between CoMFA and CoMSIA are in the use of different probes and interaction-calculating functions. In CoMFA, only probes representing steric and electrostatic interactions can be used. In CoMSIA, probes representing hydrophobic and hydrogen bond interactions, in addition to CoMFA probes may be selected. In addition, CoMSIA uses a Gaussian-type function for calculating prober-molecule interaction. By using such a smooth function, the result value is more reasonable than the function used in CoMFA, and defining a cut-off limit to remove invalid values is no longer required. The descriptors used in CoMMA are generated by computing the spatial moments of the molecules. On the other hand, the fit value is the only descriptor source employed in the HypoGen model generation process. The fit value describes the goodness of alignment between a ligand and a pharmacophore model and is obtained from a pharmacophore model generated and optimized using known structure and activity data.

The model must then be validated before it can be used to predict activity. There are some popular methods used to validate a QSAR model [56], including internal validation approaches (such as the “leave-one-out” or “leave-n-out” cross validation methods [57]), and external validation approaches. In cross validation, one (leave-one-out) or more (leave-n-out) ligand of the training set is excluded. The excluded data is predicted by the model constructed with reduced training set data. These steps are repeated until all data has been excluded and predicted, and the power of a model is determined by the accuracy of prediction [57]. External validation is a widely used method, and is considered important in the QSAR building pipeline [58]. In external validation, the capability of the model is tested using data which is not included in the training set, in contrast to internal validation, which utilizes data taken from the training set to validate the model [59]. In most of the studies, both internal and external validations are performed to ensure the reliability of the model (see following subsection “Applications of QSAR”). After the model has passed these strict validation tests, it can be used to predict the activity of novel molecules.

### 3.2. Applications of QSAR

As previously discussed, QSAR is widely used when a predictive model is required. In the work of De Melo, a PC (principle component) -SAR model was developed to predict if a molecule is active, and a PLS (partial least squares) -QSAR model was developed to predict the degree of activity of an active compound [37]. The author validated the QSAR model employing both internal (the “leave-n-out” method) and external validation approaches. From the two models, the author found several important chemical properties that contributed to the activity of the molecule.

Another example of this type of method is the QSAR analysis of CCR2 inhibitors, by Saghaie *et al.* [60]. CCR2 is a chemokine G-protein coupled receptor important in certain inflammatory disorders. In this study, the authors used a genetic algorithm to select key descriptors, and then built the QSAR model using a stepwise multiple linear regression approach. The model was validated in accordance with the literature [58] both internally (using the “leave-one-out” and “leave-five-out” methods) and externally. However, no bioassay data were given in this study.

In a study conducted by Obiol-Pardo *et al.* [61], two QSAR models were incorporated into a multiscale simulation system which was designed to predict drug-induced cardiotoxicity by the interactions of drug molecules with two potassium channels, hERG and KCNQ1. First, a docking simulation was executed to determine the active conformation of the ligands. QSAR models, which used field-based descriptors, were constructed for each potassium channel by the partial least squares method. The QSAR models were validated internally (with the “leave-one-out” method) and externally after model construction. The IC_50_ values for drugs acting on hERG and KCNQ1 predicted by the QSAR models were used as the input for two previously studied models, which could predict action potential duration and pseudo-ECG (for extraction of the QT interval) respectively. Drug-induced arrhythmia was reported to affect the QT interval and action potential duration. By employing this multiscale model, the cardiotoxicity of a novel drug was assessed without using any *in vitro* or *in vivo* assays.

## 4. Pharmacophore Models of ATP Synthase Beta Subunit-Binding Ligands

In our previous research, ATP synthase was discovered to be over-expressed on the cell membranes of a breast cancer cell line [62]. Aurovertin B, which binds to the beta subunits of ATP synthase, was also found to be a drug of potential value in the targeted therapy of breast cancer [62]. However, because of the structural complexity of aurovertin B, which makes it a difficult compound to synthesize, it has become important to design a new drug to target ATP synthase for use in cancer research. To achieve this goal, the common backbone, a substructure bearing required functional groups of the ligands, should be deduced. Although a shared backbone might be found by aligning all the molecules, important groups might not be inferred from such an alignment. The backbone and important functional groups are both vital to the design of a new inhibitor. For this reason, a pharmacophore model was built to determine common functional groups. After relevant functional groups are identified, shared backbone structure can be deduced from original structure by removing branches which contain no necessary functional groups if the molecules can be soundly aligned to the pharmacophore and each other.

**Figure 1.**
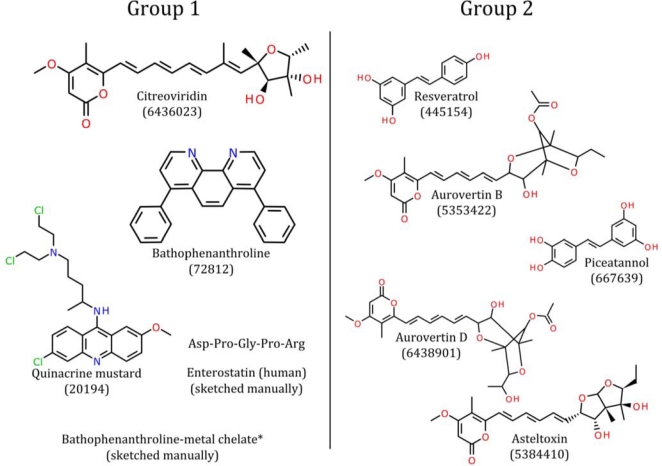
ATP synthase beta subunit ligands used in the pharmacophore modeling process. Structures, names, and PubChem Compound database IDs are listed. * Bathophenanthroline-metal chelate was constructed from bathophenanthroline and ferrous iron in the ratios 1:1 and 3:1, but a conformer could not be generated (as stated in the following paragraph). Since the conformer generation of the chelate failed, it was not included in the training set when building the pharmacophore model.

Data pertaining to known ligands of the ATP synthase beta subunit were retrieved from the PubChem [63] database by querying with their names found on the literature [64], with the exception of the peptide enterostatin and bathophenanthroline-metal chelates (see Figure 1). These two molecules were sketched manually and checked carefully using the Discovery Studio package. The ligands were separated into two groups, Group 1 (G1) and Group 2 (G2), according to their binding site location as described in the literature [64]. The compounds of G2 are known to bind to the same site on the ATP synthase beta subunit, whereas the G1 molecules are known to bind to the ATP synthase beta subunit, although the precise location of their binding site(s) was not clear: molecules in this group may bind to different location of ATP synthase beta subunit. Because reliable activity data for some molecules could not be obtained, we decided to generate a common feature pharmacophore model instead of a 3-D QSAR pharmacophore model.

Common feature pharmacophore models for each group were constructed using the HipHop algorithm [65] after the conformers were generated using the “BEST” algorithm [66]. All the algorithms are included in the Discovery Studio package (version 2.1) from Accelrys.

**Figure 2.**
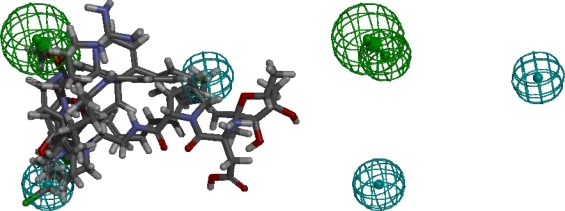
The pharmacophore model generated from the G1 molecule set. Left panel: pharmacophore model and aligned ligands; Right panel: pharmacophore model only; Green indicates a hydrogen bond acceptor feature; Cyan, a hydrophobic feature. The direction of the hydrogen bond acceptor (shown with arrows) indicated an optimal alignment of the features.

The best pharmacophore model of the G1 set, as determined by the ranking score generated by the HipHop algorithm, is shown in Figure 2. As described in previous discussions, since the molecules in G1 did not share a common binding site on ATP synthase beta subunit, they should not be used as training sets when building pharmacophore models. However, we would like to demonstrate that pharmacophore models can be built by pharmacophore elucidating software even if the training set cannot meet the criteria discussed above. Bathophenanthroline-metal chelate is not present in the alignment of the final result. The conformation generator could not generate conformers for the chelate mixture, thus the chelate was automatically excluded from the input training set. Despite the fact that G1 molecules are not known to have a common binding site, a pharmacophore model could be generated by HipHop, which was an unexpected finding. The ligands might share the same binding site on the ATP synthase beta subunit because they share some chemical features, as indicated by the generated pharmacophore model; further structural studies of ATP synthase are required to reveal the binding site(s) of these ligands. Nevertheless, researchers need to shoulder the responsibility of checking the binding characteristics of ligands, as described in the previous section; otherwise a pharmacophore model which stands a high chance of being meaningless may be generated, as demonstrated by the pharmacophore model presented here.

**Figure 3.**
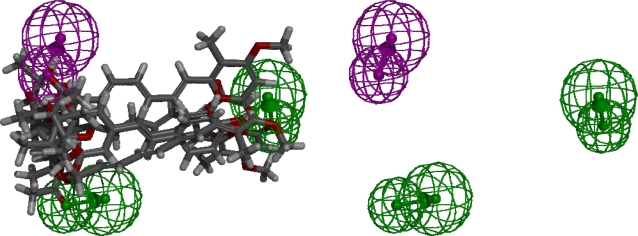
The pharmacophore model generated from the G2 molecules. Left panel: the pharmacophore model and the aligned ligands; Right panel: pharmacophore model only; Green indicates a hydrogen bond acceptor feature; Purple, a hydrogen bond donor feature. The direction of the hydrogen bond acceptor and hydrogen bond donor (shown with arrows) indicates optimal alignment of the features.

The best pharmacophore model of the G2 set, as determined by the ranking score generated by the HipHop algorithm, is shown in Figure 3. As predicted, the G2 molecules were aligned better than the G1 molecules. Three similar molecules, aurovertin B, aurovertin D, and asteltoxin, were in this group. In addition, resveratrol and piceatannol are two very similar molecules. However, it can be clearly seen that the G2 molecules can be separated into two sets by putting resveratrol and piceatannol together into one group, and putting the remaining molecules into the other. Although this action might affect the generated pharmacophore model, the results bear some resemblance to the results of the docking simulation performed in our previous study [62]: The hydrogen bond donor feature can be mapped to the Lue342 residue, and the hydrogen bond acceptor features can be mapped to the Lys382, Arg412 residues of the ATP synthase beta subunit. These observations were not beyond what we expected to achieve, since a 3-D pharmacophore model can reflect the arrangement of the key residues of the binding site [19]. However, after careful inspection, the pharmacophore model feature vectors indicating the direction of the hydrogen bonds were different from those of the previous docking simulation, and the interaction poses were different as well. In addition, the fit value (not shown) of aurovertin B was not as high as expected. As in our previous research, the activity of aurovertin B was significantly higher than resveratrol and piceatannol [62]. Furthermore, accurate alignment of the two aurovertins and asteltoxin was expected because of their structural similarity but was not observed in our results. Although this pharmacophore model can reflect the binding mode discovered in our previous research, this model was not validated experimentally. We present the model here for demonstration of the pharmacophore modeling process. One should conduct validation before using this pharmacophore model in their research.

## 5. Conclusions

QSAR and pharmacophore models are two widely used techniques in the rational drug design process. In this review, the basics of the pharmacophore and QSAR techniques were introduced, and the basic modeling processes for both methods, along with some possible pitfalls, were discussed. After the fundamental concepts of the two methods were covered, some studies using each technique were explored. Previously unpublished pharmacophore models generated from a range of ATP synthase beta subunit ligands were also used to demonstrate the workflow of a pharmacophore modeling process. It is important to know the basics and pitfalls of these computer-aided drug design techniques. Knowledge of such techniques can aid researchers in selecting the best technique for their given objectives. However, results may still be prone to error, especially when there is disagreement between input and assumptions of the selected techniques. It is also important to bear in mind that better results may be yielded by combining QSAR and pharmacophore models with other drug design methods, as shown in the example sections of this review.
